# Effects of 8-week core training on core endurance and running economy

**DOI:** 10.1371/journal.pone.0213158

**Published:** 2019-03-08

**Authors:** Kwong-Chung Hung, Ho-Wa Chung, Clare Chung-Wah Yu, Hong-Chung Lai, Feng-Hua Sun

**Affiliations:** Department of Health and Physical Education, The Education University of Hong Kong, Hong Kong SAR, China; University of Tennessee Health Science Center College of Graduate Health Sciences, UNITED STATES

## Abstract

The purpose of this study was to examine the effects of 8-week core training on core endurance and running economy in college athletes. Twenty-one male college athletes were randomly divided into 2 groups: a control group (CON) (n = 10) and a core training group (CT) (n = 11). Both groups maintained their regular training, whereas CT attended 3 extra core training sessions per week for 8 weeks. The participants were assessed before and after the training program using sensory organization test (SOT), sport-specific endurance plank test (SEPT) and 4-stage treadmill incremental running test (TIRT). Compared with the pre-test, significant improvements were observed in post-test SOT (78.8 ± 4.8 vs. 85.3 ± 4.8, p = 0.012) and SEPT (193.5 ± 71.9 s vs. 241.5 ± 98.9 s, p = 0.001) performances only in CT. In the TIRT, the post-test heart rate values were lower than the pre-test values in CT in the first 3 stages. In stage 4, the post-test oxygen consumption (VO_2_) was lower than that in pre-test in CT (VO_2_: 52.4 ± 3.5 vs. 50.0 ± 2.9 ml/kg/min, p = 0.019). These results reveal that 8-week core training may improve static balance, core endurance, and running economy in college athletes.

## Introduction

Core muscles have been suggested not only to protect the spine from excessive force, but also to play an important role in body stabilization and force generation during sporting activities [1]. Core training has become a common exercise in rehabilitation as well as in fitness. For example, research has shown that core training can facilitate recovery from injury and relieve chronic lower back pain [2–4].

Core stability, strength and endurance are the most important core abilities that ensure spine stability for force production and injury prevention [5]. Core stability refers to the stability of the spine [5] and determines the efficiency of biomechanical function for maximizing force generation. Okada et al. [6] investigates the relationship between core stability and functional movement and performance. The results show that core stability is not a strong indicator of functional body movement (as tested by functional movement screen) or sports performance (as tested by backward medicine ball throw, T-run, and single-leg squat). Core strength refers to the muscular ability to stabilize the spine through contractile forces and intra-abdominal pressure, actively controlling spine stability through coactivation of the trunk muscles [5]. Excessive core strength, however, is suggested to cause a greater core instability [5].

Core endurance is the most crucial component in core training [5] because it supports core muscles in maintaining an efficient trunk position. Barati et al. [7] indicates that core endurance is important to spinal stability during prolonged exercise. Koblbauer et al. [8] suggests that a positive relationship exists between core endurance and running kinematics. Tong et al. [9] indicates that a high intensity maximum run might induce the core muscle fatigue. Therefore, improving core endurance may benefit running performance.

Running economy is a controversial topic in core training. Studies have shown that core function is related to running kinematics and respiratory work [8, 10, 11]. Core muscles allow optimal force production, to control, support, and move extremities [1]. Proper core exercise may result in an improvement in core endurance, respiration, and movement efficiency [12]. However, not all studies have supported these findings. For example, a study revealed that 6-week core training had no significant improvement in maximal oxygen consumption (VO_2max_), running economy, or running posture [13]. Sato et al. [14] observed a significant improvement in running performance after core training, but no improvement in running kinetics was noted. More recently, Tong et al. [10] showed greater improvements in core endurance, running economy, and running performance after 6-week core training. The inconsistency of the findings of these studies may stem from differences in core training programs, core training durations, or even participants. Because of limited studies and controversial findings in this topic, the aim of this study was to investigate the impact of 8-week core training on core endurance and running economy. The hypothesis was that 8-week core training may improve core endurance and running economy.

## Materials and methods

### Participants

A total of 25 male college athletes with at least 3 years of regular sports training experience (generally 3 days per week) were recruited from different university sports teams including long-distance run, football, basketball and rugby. Among the 25 participants, only 21 participants (CON: n = 10; CT: n = 11) completed the post-test session and only data from these participants were included for data analysis. Injury during the experimental period caused 4 participants to withdraw. Female participants were excluded because of the potential influence of menstruation. All participants received a briefing with written and verbal information about the study protocol and possible risks. A consent form and physical activity readiness questionnaire were signed and completed before the experiments. Participants were requested to maintain their habitual dietary intake, lifestyles, and training models. Vigorous exercise, alcohol, and caffeine were restricted 48 hours before laboratory tests. Participants were instructed not to eat for at least 2 hours before each test. Ethical approval of this study was obtained from the Human Research Ethics Committee of the Education University of Hong Kong.

### Experimental design

Participants with matched physical characteristics and training background were randomly assigned to 2 groups: a control group (CON) and a core training group (CT) (Table 1). In order to find out the effectiveness of the core training intervention on the core endurance and running economy, participants in CT completed 8-week core training, which included the fundamental strength phase (week 1 to 3), the stabilization phase (week 4 to 6) and the functional strength phase (week 7 to 8). Apart from the additional core training, all participants maintained their own regular training. During the 8-week period, participants did not receive other core training or specific running training. To monitor the training status of the 2 groups throughout the 8-week period, all participants were required to complete a training log to record their daily training hours and activities.

**Table 1:** Physical characteristics and training background of participants in CON and CT.

	CON (n = 10)	CT (n = 11)
Height (cm)	174.0±4.3	176.0±7.2
Weight (kg)	69.0±6.3	65.9±4.8
VO_2max_ (ml/kg/min)	56.4±5.4	58.3±4.7
Training hours per week (hrs)	11.3±6.2	9.5±3.6

Values are mean ± SD; CON: control group, n = 10; CT: core training group, n = 11; No significant difference in any variable between CON & CT (p>0.05).

### Procedure

**Fig 1:**
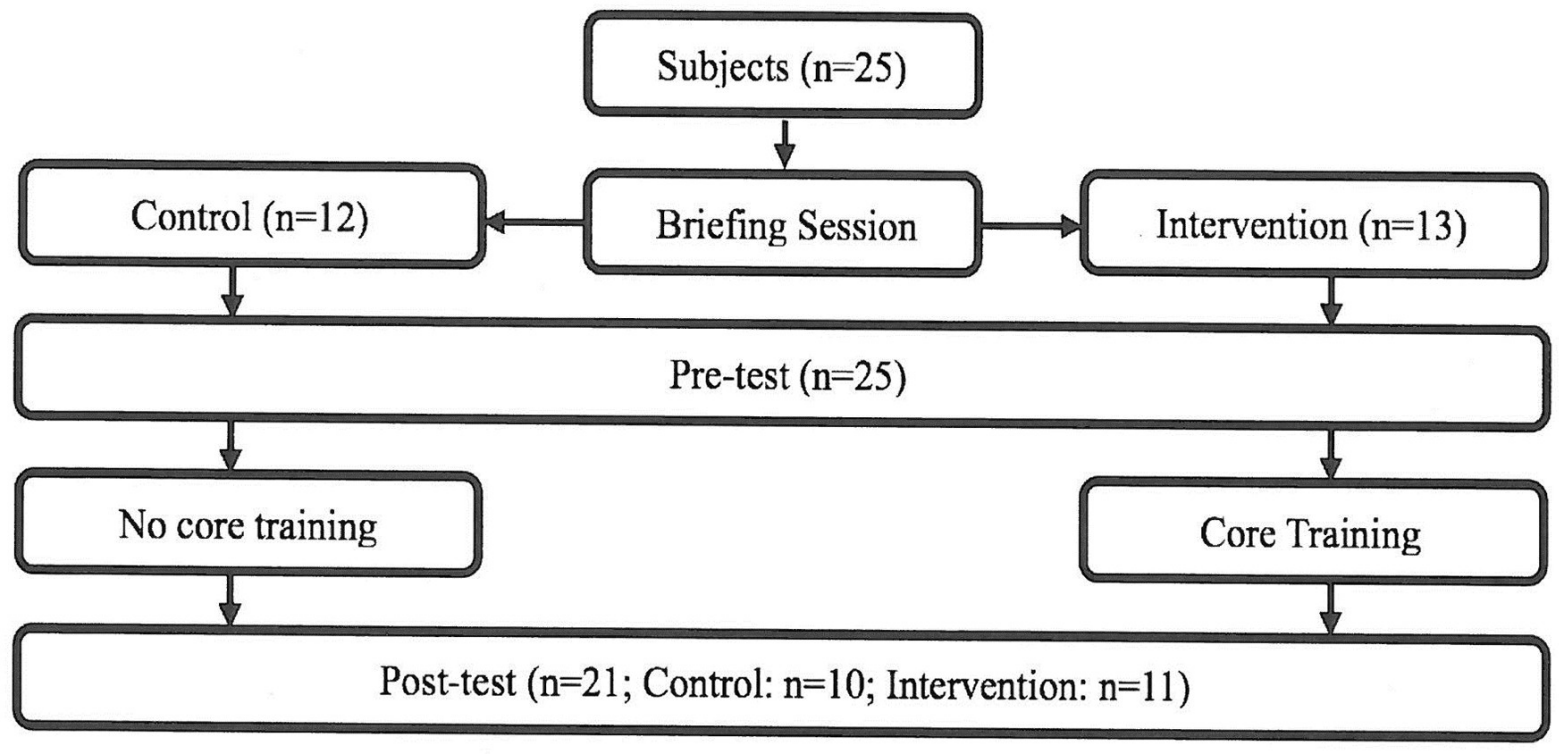
Flow chart of experiment.

The flowchart of the experiment is presented in Fig 1. All participants were required to attend a briefing session before the pre-test. In both pre- and post-test sessions, the participants were required to complete a sensory organization test (SOT), sport-specific endurance plank test (SEPT), and treadmill incremental running test (TIRT) in the laboratory. Both groups followed the same test order: the SOT, followed by the SEPT and then the TIRT. To minimize the impact of fatigue on performance, participants took a 5-minute rest after the SOT and a 15-minute rest after the SEPT. All participants are required to get familiar with the procedures and requirements of all these tests before the pre-test session.

The SOT was conducted using computerized dynamic posturography (CDP; Smart Equitest, NeuroCom International Inc., Clackamas OR, USA). The CDP can identify and quantify sensory (visual, vestibular, and somatosensory) and motor functions to assess static balance control [15]. Static balance is strongly correlated with core endurance [7]. The SOT has acceptable test-retest validity and reliability (intra-class correlation (ICC) was 0.35 to 0.79 in 6 sessions) and is used to test sensory organization and balance control in amateur athletes [16, 17].

During the SOT, all participants stood barefoot on a dual force plate (46 cm × 46 cm). A harness was provided for safety. The step width during standing was standardized according to the participants’ height. They were then subjected to 6 conditions of visual and supporting surface perturbations. Conditions 1, 2, and 3 included eyes open, eyes closed, and eyes open in a sway-referenced visual surround, respectively. Conditions 4, 5, and 6 were performed on a sway-referenced platform with eyes open, eyes closed, and eyes open in a sway-referenced visual surround, respectively. Each condition was tested 3 times and each test lasted 20 seconds. After the test, an equilibrium score was obtained for each trial and overall performance. The overall equilibrium score represented the balance performance of participants.

Before SEPT, all participants were demonstrated the correct plank posture, i.e., the body is supported on palms, elbows, and toes with the spine in a neutral position while keeping the head, torso, and legs aligned [11]. The SEPT included 4 variations of plank: basic plank, alternated arm lift, alternated leg lift, and cross arm leg lift. The stages of the SEPT were as follows: (A) Hold the basic plank for 60 seconds; (B) lift the right arm off the ground for 15 seconds; (C) lower the right arm and lift the left arm for 15 seconds; (D) lower the left arm and lift the right leg for 15 seconds; (E) lower the right leg and lift the left leg for 15 seconds; (F) lift the left leg and right arm off for 15 seconds; (G) lower the left leg and right arm and lift the right leg and left arm for 15 seconds; (H) return to the basic plank for 30 seconds; and (I) repeat stages (A) through (H) until the participants fail to maintain the plank posture. During the SEPT, surface electromyography (sEMG) was used to measure the degree of core muscle activation in the rectus abdominis (RA), external oblique (EO), and the erector spinae (ES). Electrodes were placed according to a previous study [18]. One pair of electrodes was placed 3 cm lateral to the umbilicus for the RA. One pair of electrodes was placed midway between the anterior superior iliac spine and the lowest ribs for the EO. The electrodes for the ES were placed 2 cm lateral to the L4-L5 interspace. An sEMG recorder (BTS FREEEMG 300, BTS Bioengineering Corp., Brooklyn, NY, USA) was used to record the sEMG signal. All EMG signals were collected at 1000 Hz and filtered by using standard band-pass filtering techniques with cutoffs of 20–350 Hz. Raw EMG signals were processed and analyzed with the Myolab 2.2 system software (BTS, MI, Italy). During data processing, the EMG signals were full-wave rectified and processed by root mean squared algorithm. The average EMG values were analyzed each 15 seconds during the SEPT.

The TIRT consisted of an incremental VO_2_-speed test and a VO_2max_ test. The treadmill grade was set to 1%, because the oxygen cost associated with a 1% treadmill grade is similar to that associated with running outdoors at velocities between 10.5 and 18.0 km/h [19]. The participants started the VO_2_-speed test with a 6-minute warm-up at 8.0 km/h. They then ran for 4 incremental stages at speeds of 7.0, 9.0, 11.0, and 13.0 km/h. Each stage lasted 4 minutes. After the incremental test, a 10-minute rest was provided to prepare for the VO_2max_ test. In the VO_2max_ test, the participants began at 10.0 km/h and increased the speed at a rate of 1.0 km/h per minute until reaching 13.0 km/h. When the speed was set at 13.0 km/h, the treadmill grade was increased by 1% per minute until VO_2max_ was reached. The VO_2max_ value was determined by 3 criteria: (A) Respiratory Exchange Ratio > 1.1; (B) Heart Rate (HR) ± 10 age-estimated HR_max_; and (C) failure of participant to continue. All ventilation parameters and pulmonary gas exchange were gauged using a Cortex ergospirometry system (pre-test: METAMAX 3B; post-test: METALYZER II, Germany). The collected data were adjusted to 30 seconds for calculation.

Blood lactate concentration (LA), HR and rate of perceived exertion (RPE) were measured within 30 seconds by the end of each stage of the TIRT. LA was measured by portable lactate analyzer (Lactate Plus Meter, Nova Biomedical, MA, USA). HR was measured by an HR monitor (H7 heart rate sensor, Polar, Finland). Creatine kinase (CK) was collected after the TIRT and measured by a chemical analyzer (Reflotron, Oberoi Consulting Ltd, UK).

In general, core training programs should include training 2 to 4 times per week for 4 to 8 weeks [20, 21]. Variations of plank, crunch, and trunk twist are commonly used as core exercises. Although the core functions in all sports is similar, i.e., the force generation/ transfer and body stabilization, no consensus is available regarding what is the most effective core exercise protocol to improve core functions. The core training program in this study consisted of 3 parts: fundamental strengthening (3 weeks), stabilization (3 weeks), and functional strengthening (2 weeks). CT had 3 training sessions per week, with 2 sessions in the lab and 1 session at home. Each session included 4 exercises and lasted 30 minutes. All sessions were instructed by a certified personal trainer. Mini-bands, air pads, and BOSU balls assisted in certain phases of core training. Home exercise required participants to hold a prone plank 3 times for 1 minute each. Details of the training program design are shown in Table 2.

**Table 2:** Training program design.

Exercise Protocol	Reps/Time	Assisted Equipment	Sets
**Warm-up Exercise**			
Dead-bug Exercise	30	Mini-band	1
**Week 1 to 3 (Fundamental Strength)**			
Crunch	20(week1-2) /25(week 3)	/	3
Back Bridge	20(week1-2) /25(week 3)	/	3
Plank	45s (week1-2) / 60s (week 3)	Mini-band	3
Side Plank with leg raise	30s (week1-2) / 45s (week 3)	/	3
**Week 4 to 6 (Stabilization)**			
Crunch	25	Air pad	3
Split Leg Bridge	20 per side	/	3
Plank with AP/BOSU and band	60 seconds	Mini-band & BOSU	3
Side Plank with leg raise	30 seconds	Air pad	3
**Week 7 to 8 (Functional Strengthening)**			
Mountain Climber	40	Mini-band	3
Pallof Press	60 seconds	BOSU	3
Split Leg Bridge	25	Air pad	3
Plank Variation	30s per type (5 types)	/	2

*AP: Air Pad; BOSU: BOSU Balance Trainer

The primary goal of the fundamental strength phase was to increase basic core strength, and the training focused on core muscle activation and motion control. The focus of the stabilization phase was core and body stabilization. The level of difficulty was increased with unstable surfaces or unilateral movement. In the functional strengthening phase, the training program followed the definition of functional training, encompassing a range of methods to help apply training to competition or specific function [22], and focused on anti-motion of core function [23].

### Statistical analysis

All data were presented as mean ± SD, and were analyzed using IBM SPSS Statistics (version 21, IBM Corp., NY, USA). Normality was checked using the Kolmogorov-Smirnov test, and equal variance assumptions was checked by Levene test. For baseline physical characteristics, an independent t-test was used to compare the difference between groups. Crossover data for repeated measures were analyzed by two-way (Group × Time) repeated measures analysis of variance (ANOVA). A Bonferroni post hoc test was applied to determine the difference between groups or within-group changes from pre-test to post-test. Significant level was defined as p ≤ 0.05.

## Results

No difference was observed between the 2 groups (CON vs. CT) in height (174 ± 4.3 vs. 176 ± 7.2 cm; p = 0.386), weight (69.0 ± 6.3 vs. 65.9 ± 4.8 kg; p = 0.125), VO_2max_ (56.4 ± 5.4 vs. 58.3 ± 4.7 mL/kg/min; p = 0.193), or SOT performance (80.3 ± 7.1 vs. 79.1 ± 4.7; p = 0.899) before the intervention. Additionally, no difference was noted between the 2 groups (CON vs. CT) in the training hours per week (11.3 ± 6.2 vs. 9.5 ± 3.6 h; p = 0.427) excluding core training in CT.

### Treadmill test

Table 3 presented a summary of the data from the TIRT. No interaction effect (group and time) was observed for HR, LA, RPE, or VO_2_ values. In CT, the observed HR values in the post-test were lower than those in the pre-test for the first 3 stages (ST1: p = 0.009; ST2: p = 0.006; ST3: p = 0.027). In CON, only the HR value during stage 1 was lower than that in the pre-test (p = 0.049). Moreover, in CT, the post-test VO_2_ value in stage 4 decreased, compared with the pre-test value (p = 0.019). However, in CON, no difference in VO_2_ value was found between the pre-test and post-test at any stages. Compared with pre-test data, the post-test LA values increased in stage 1 in CT but decreased in stages 3 and 4 in CON. The only difference in RPE between the pre- and post-tests was found in stage 2 in CT.

**Table 3:** Summary of the result on treadmill test.

	Pre-training Test					Post-training Test				
Stage:	1	2	3	4	VO_2max_	1	2	3	4	VO_2max_
VO_2_ (ml/kg/min)										
CON	30.25±2.82	37.71±2.36	42.13±5.22	47.63±5.48	56.40±5.42	29.36±1.9	37.81±2.26	43.06±2.04	47.39±3.04	53.60±4.64
CT	30.12±3.22	38±3.11	46.25±3.8	52.38±3.54	58.27±4.73	32.65±4.34	37.80±2.94	46.45±5.32	49.98±2.89*	55.73±5.11
HR (bpm)										
CON	136.4±15.2	153.8±10.9	171.2±11.9	182±9.3	192.5±8.9	124.9±17.7*	150.8±10	169.5±9.8	181.3±5.8	193.2±6.7
CT	142.3±14.6	161.6±15.8	176.1±14.2	185±14.9	194.1±9.4	132.2±13.9*	151.8±14.5*	170.3±14.8*	182±13.2	193.3±7.2
RPE										
CON	1.7±0.95	3±1.25	4±0.94	6.6±1.71	/	1.3±0.92	3.4±0.69	4.7±1.34	6.3±2.11	/
CT	0.7±0.54	2.1±1.17	3.7±1.57	6.4±2.32	/	1±0.91	2.8±1.7*	4.7±2.31	6.9±2.23	/
LA (mmol)										
CON	2.27±0.99	2.75±1.23	4.36±1.31	7.91±2.59	12.52±2.24	2.41±0.95	2.3±0.92	3.84±1.23*	6.65±1.77*	12.02±2.72
CT	1.83±0.54	2.37±0.7	4±1.45	7.07±2.63	10.76±2.45	2.44±0.78*	2.45±1.11	4.09±1.93	6.93±3.29	10.84±1.57

CON: control group, n = 10; CT: core training group, n = 11.

* p<0.05 pre-test vs. post-test

### Sensory organization test

**Fig 2:**
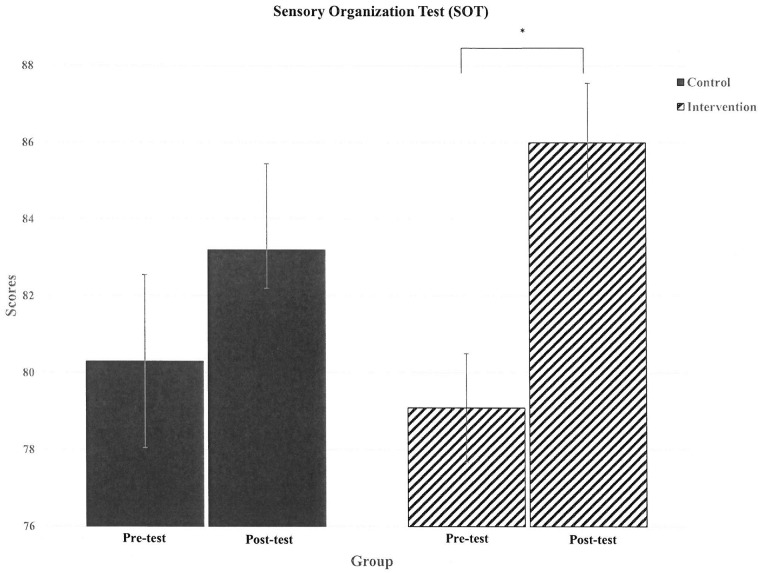
The change of SOT scores in two groups. SOT: Sensory Organization Test; CON: control group, n = 10; CT: core training group, n = 11. * p<0.05 pre-test vs. post-test.

The mean SOT score increased in CT after 8 weeks of core training (pre-test vs. post-test:78.8 ± 4.83 vs. 85.3 ± 4.83, p = 0.02, Fig 2), but not in CON (pre-test vs. post-test: 80.3 ± 7.12 vs. 83.2 ± 7.08, p = 0.184). However, no interaction effect or group effect was observed in two-way repeated measures ANOVA (F = 2.35; p = 0.16).

### Sport-specific endurance plank test

**Fig 3:**
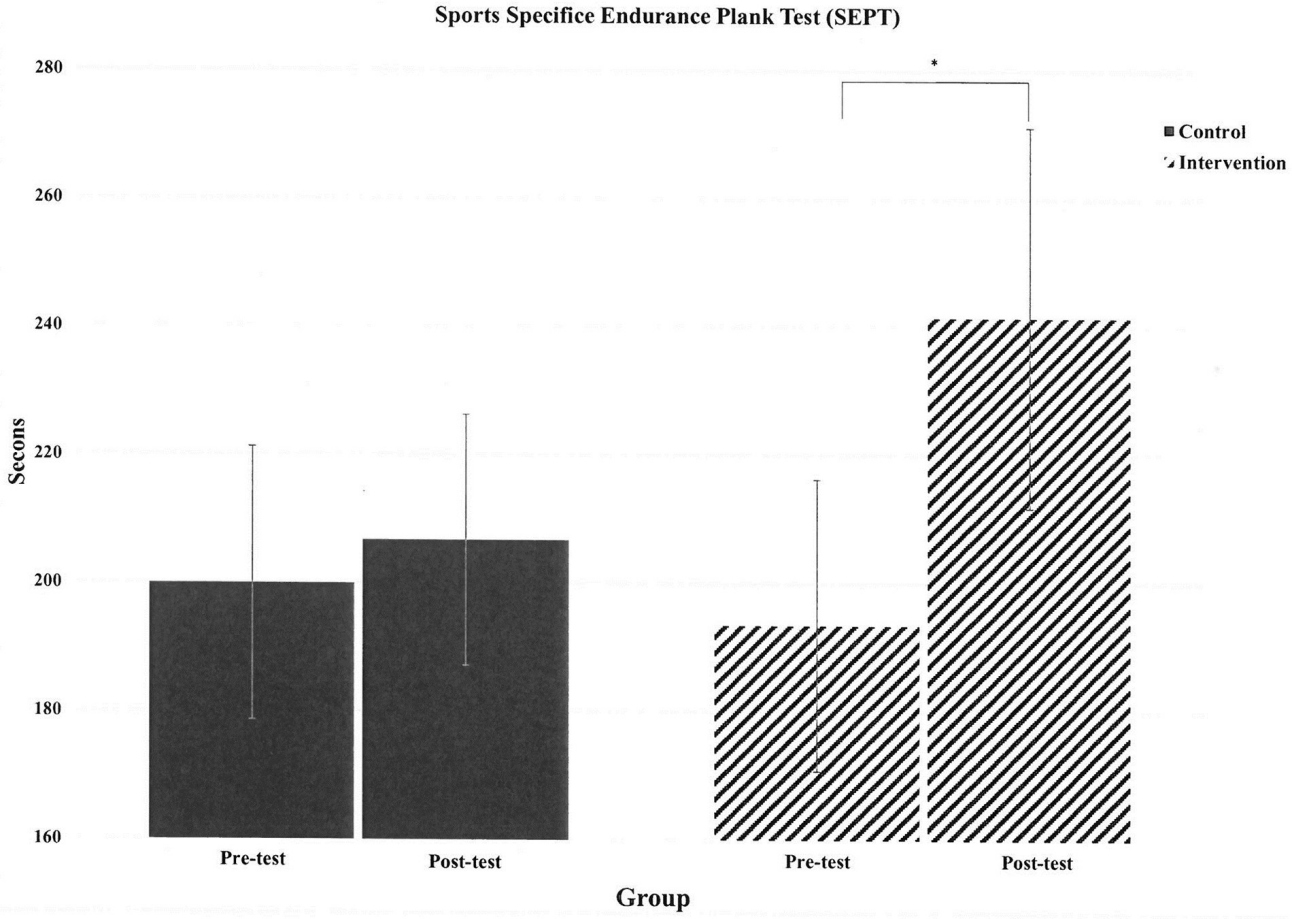
The change of SEPT scores in two groups. SEPT: Sport-specific Endurance Plank Test; CON: control group, n = 10; CT: core training group, n = 11. * p<0.05 pre-test vs. post-test.

The results revealed no differences in the SEPT score (Fig 3) or sEMG data between the 2 groups in 2-way repeated measures ANOVA. Furthermore, no interaction effect (group and time) was noted (F = 4.014; p = 0.076). Compared with pre-test data, the mean post-test SEPT score increased in CT after 8 weeks of core training (pre-test vs. post-test: 193.5 ± 71.9 vs. 241.5 ± 93.9 s, p = 0.01), but not in CON (pre-test vs. post-test: 199.9 ± 67.3 vs. 206.7 ± 61.9 s, p = 0.720). The sEMG data indicated that the right (pre-test vs. post-test: 0.0145 ± 0.0118 vs. 0.0321 ± 0.0387 μV, p = 0.005) and left ES (pre-test vs. post-test: 0.0197 ± 0.0202 vs. 0.0327 ± 0.0370 μV, p = 0.044) were more activated in the post-test than in the pre-test in CT. No difference was found in Right RA (pre-test vs. post-test: 0.0868 ± 0.091 vs. 0.0892 ± 0.091 μV, p = 0.85), Left RA (pre-test vs. post-test: 0.0664 ± 0.0556 vs. 0.0823 ± 0.0659 μV, p = 0.32), Right EO (pre-test vs. post-test: 0.0477 ± 0.0222 vs. 0.0585 ± 0.0278 μV, p = 0.356) and Left EO (pre-test vs. post-test: 0.0364 ± 0.0293 vs. 0.0924 ± 0.0846 μV, p = 0.085) for CT. No differences were found in all sEMG data between the pre- and post-tests in CON.

## Discussion

The primary finding of this research is that 8 weeks of core training may improve core endurance and running economy. Compared with the pre-test, several indicators including VO_2_, HR, SOT score, and SEPT score were improved after core training in CT, but no differences in these indicators were observed in CON.

Running economy refers to the energy required (usually inferred from VO_2_) to maintain a constant speed of running. In this study, the TIRT is adopted to measure the difference in energy expenditure after 8-week core training for the same submaximal exercise, as energy expenditure and oxygen consumption can accurately reflect energy metabolism [24]. Two identical treadmill test protocols for pre- and post-training were applied in both groups, therefore, changes in VO_2_ may reflect changes in running economy. This study found that the post-test VO_2_ value in stage 4 was lower than that in the pre-test in CT (Table 3). When comparing the pre-test VO_2_ values in CON and CT, the percentage differences in post-test VO_2_ value in stage 4 were -0.5% and -4.6%, respectively. This indicates that running economy improved after the 8-week core training program adopted in this study. This finding is consistent with that of a previous study, in which running economy was significantly improved at the speed of the onset of blood lactate accumulation for treadmill running after 6-week core training [10]. Although decreased VO_2_ was found only in stage 4 (around 90% VO_2max_) in CT, the HR values for the first three stages were lower in the post-test than in the pre-test in this group, which may indicate the improvement in running economy for low- to moderate-intensity exercise. Notably, VO_2max_ did not change significantly after intervention in either group, which is consistent with the finding of a previous study by Stanton et al. [13], who observed no improvement in VO_2max_ after 6-week core training. These findings suggest that 6–8 weeks of core training may not increase VO_2max_, but it may improve the running economy which may benefit running performance. Previous research also suggested that core muscles are used to optimize running economy during submaximal intensity exercise [1]. Additionally, because of training effect including better connection of lower extremities and upper extremities [1, 20], the core muscular strength may be improved after 8-week functional strength training in the present study. The improved core muscular strength may also be explained by the improvement in intermuscular coordination after the functional strength training [25]. Therefore, the two main functions of core muscle–force transference and body stabilization–may be improved, so as to improve the mechanical efficiency and benefit the running economy. It should be noted that, although the duration of core training programmes were similar between our study (8 week) and previous studies (6 weeks), the participants and the training protocols were different among these studies. In one study [10], recreational adult runners were recruited and simultaneous inspiratory muscle training program was adopted. Whereas in another study [13], young athletes were participants and Swiss ball were used in core training programme. Different training programmes may affect the final results, e.g., stabilization exercise has been suggested to have specific training effect on static and dynamic balance, Cooper’s test, and rebound jump, compared with conventional trunk exercise [26]. Therefore, it seems that more studies are still needed before drawing a strong conclusion.

Another purpose of the present study was to investigate the effect of 8-week core training on core endurance. The SEPT is a valid, reliable, and practical method for assessing core endurance among athletes, because it has excellent test-retest reliability (ICC: 0.97) [18]. This test has also been shown to be correlated with Yo-Yo intermittent recovery test, Cooper test, and agility test in adolescent soccer players [27]. The SEPT performance was improved in CT but not in CON (Fig 3), indicating the improved core endurance after core training. The sEMG data also suggested that more muscular activities were induced in the ES during the SEPT in the CT group after the core training program, although similar result was not observed in the RA and EO in the present study. A previous study revealed that 55%-58% of muscle fibers in the abdominis are type I fibers which are associated with muscular endurance [28]. Therefore, it is possible that core training specifically improve core endurance. However, this may not be caused by increased abdominal muscular activities. Research suggested that poor core endurance may negatively affect running kinematics, and that the inability to maintain trunk position increases lower extremity loading during long-distance running [8]. Moreover, Tong et al. [10] investigated the potential effect of core muscle fatigue during high-intensity running and found that core muscle fatigue strongly impaired high-intensity running (85% VO_2max_) performance. Therefore, improving core endurance through regular core training is crucial for runners.

Two considerations were made in designing the core training program: (A) building stable core muscles and improving core endurance, and (B) establishing core muscle motor control to provide a sufficient base for force generation and transfer. Creating sufficient spinal stability depends on not only muscular force improvement, but also on stabilizing techniques such as abdominal bracing, which may take advantage of the leverage afforded by the movement arm [11]. In a comprehensive program, unstable surfaces should be included because spinal stability is required for efficient execution of physical tasks [28, 29]. Therefore, this study adopted a core training program that prescribed the dead-bug warm-up exercise, which can activate almost all global core muscles [30], and used unstable surface equipment to increase core muscle activation for core stabilization [31]. In addition to SEPT performance, the SOT score was improved after core training in CT, but not in CON (Fig 1). Effective core muscle function may reduce excessive limb motion during exercise, because proximal core activation enhances the efficiency of distal segment function. This may be caused by the higher precision and stability of distal extremities. Core muscles provide single-joint segmental stabilization, which allows multi-joints muscles to work more efficiently to control spine motion [32]. Therefore, these results suggest that the core training program used in the present study benefits both body balance and core endurance.

There are several limitations in the present study. Firstly, the absolute values were used for the sEMG data without normalization by maximal voluntary contraction (MVC), which may slightly affect the reliability of the sEMG data. Secondly, only male participants from several different college sports teams were recruited. Thirdly, Kinetics and kinematics were not investigated during running which may limit the application of the present study. Fourthly, some participants withdrew from the study because of injury. Additionally, one unexpected limitation occurred during this study. The METAMAX 3B sensor failed during the experiment. A similar spirometer, METALYZER II, was substituted for the METAMAX 3B. Despite the abovementioned limitations, the results may provide valuable information regarding the potential benefits of core training to core endurance and running economy. Future studies are still required to clarify whether core training may benefit elite athletes in core endurance and running economy, especially for female athletes. Also, more measurements on kinetics and kinematics should be included to provide more valuable information. Furthermore, the normalization should be performed to measure core muscle activities when using sEMG data.

## Conclusion

In conclusion, 8 weeks of core training may improve static balance, core endurance and running economy of male college athletes. Field tests could be performed to directly investigate the effect of core training on specific sports performance.
